# Examining the impact of a universal positive psychology program on mental health outcomes among Australian secondary students during the COVID-19 pandemic

**DOI:** 10.1186/s13034-023-00623-w

**Published:** 2023-06-12

**Authors:** Mirjana Subotic-Kerry, Taylor A. Braund, Dervla Gallen, Sophie H Li, Belinda L. Parker, Melinda R. Achilles, Cassandra Chakouch, Simon Baker, Aliza Werner-Seidler, Bridianne O’Dea

**Affiliations:** 1 Black Dog Institute, University of New South Wales, Hospital Road, Randwick, NSW Australia; 2 Faculty of Medicine and Health, University of New South Wales, Sydney, NSW Australia; 3 Orygen, University of Melbourne, Melbourne, VIC Australia

**Keywords:** Adolescent, COVID-19, Positive psychology, Anxiety, Depression, Internet

## Abstract

**Background:**

This study aimed to examine the impact of a web-based positive psychology program delivered universally to secondary school students during school closures caused by the COVID-19 pandemic in New South Wales, Australia.

**Methods:**

Using a quasi-experimental design conducted in 2020, 438 students aged 12–15 years (73% male) from 4 secondary schools were invited to complete the ‘Bite Back Mental Fitness Challenge’. This web-based program consisted of 7 self-directed modules that targeted 5 key domains of positive psychology. Self-reported symptoms of anxiety and depression and help-seeking intentions for mental health were assessed at baseline prior to school closures (February to March 2020) and at post-test after the return to school (July to August 2020). At post-test, students also reported on their perceived changes in mental health and help-seeking behavior for mental health during the pandemic. Completion of the program modules was recorded.

**Results:**

A total of 445 students consented and 336 (75.5%) completed both assessments. On average, participants completed 2.31 modules (SD: 2.38, range: 0 to 7). There was no change in symptoms of anxiety and depression or help-seeking intentions between baseline and post-test, with no significant effects for gender and history of mental illness. Students who were symptomatic for anxiety and depression at baseline reported lower symptoms at post-test, but this change was not significant. Ninety-seven students (27.5%) reported that their mental health had worsened during the pandemic, and a significant increase in anxiety and depressive symptoms was found in this subsample at post-test. Only 7.7% of students reported a change in their help-seeking behavior, with increased mental health support sought from the Internet, parents, and friends.

**Conclusions:**

The universal delivery of a web-based positive psychology program during school closures did not appear to be associated with improved mental health symptoms; however, completion of the modules was low. Different effects may emerge when selectively delivered to students with mild or greater symptoms. The findings also suggest that broader measures of mental health and wellbeing, including perceived change, are key to the mental health surveillance of students during periods of remote learning.

## Introduction

Worldwide, COVID-19 resulted in several disruptive changes to the daily lives of adolescents, including school closures, shifts to home-based learning, and home confinement, which reduced their participation in sport, work, and social activities [1–3]. These changes have been associated with heightened uncertainty, greater concerns about illness [4–6], and feelings of isolation and loneliness, all of which have been correlated with poor mental health in adolescents [2, 7]. Several datasets have shown increased rates of mental health problems in all age groups during the COVID-19 pandemic [8]. The pandemic appears to have had a greater negative impact on anxiety and depression in young people and females when compared to older age groups and males [8]. A meta-analysis of the prevalence rates of depression and anxiety symptoms in adolescents found that the incidence of these had doubled in comparison to pre-pandemic estimates [9]. Other cross-sectional research has indicated that young people with a pre-existing diagnosis or history of mental illness were at a higher risk of poorer mental health during the pandemic [10, 11], and high levels of depression, anxiety and loneliness were reported in both clinical and non-clinical populations of young people [12]. Collectively, these studies indicate that the COVID-19 pandemic has had a significant impact on the prevalence of anxiety and depressive disorders in young people, with worse outcomes found in females, younger populations, and those with pre-existing vulnerabilities.

The COVID-19 pandemic has also significantly disrupted adolescents’ access to mental health services. In the UK, one in four young people (aged 13 to 25 years) reported that they were unable to access mental health support during the pandemic [11]. In an Australian survey of 1000 psychologists, 88% reported an increase in wait times since March 2020 and 56% reported the wait period for new clients to be greater than three months [13]. The lockdowns resulted in many in-person mental health services making an abrupt, and often cumbersome, shift to telehealth [14–16], and the demand for existing telehealth services significantly increased. In Australia during the first wave of the pandemic (March 2020 to April 2020), the demand for Kids Helpline (a 24-hour free telephone, webchat and email counselling service for youth aged 13 to 25) increased 1.5 times [17]. Widespread school closures also reduced adolescents’ access to services, as many students received in-person mental health support via their school [18]. In Australia, the transition of in-person school counselling to web-based delivery was hampered by practical and ethical challenges. As a result, school counsellors reported a reduction in the number of students engaged in school counselling during the first wave of the pandemic [19]. In the US, school counsellors described the impact of school closures on their services as “chaotic and overwhelming” [20] and many reported reduced consultations with students [21]. Despite this upheaval, schools continued to play a key role in caring for students during closures as teachers remained in regular contact with students and could act to alleviate the negative impacts of heightened distress on classroom learning [22]. Schools also helped facilitate feelings of connectedness between students and their peers, which may have further protected students’ mental health [23]. Overall, the COVID-19 pandemic has prompted increased recognition and examination of the role of schools in promoting and preserving the mental health of students, particularly during times where in-person interactions are non-existent.

Digital mental health technologies (e.g. websites, web-based programs, mobile apps) may present several advantages for schools needing to address student mental health remotely. These technologies can improve young people’s access to evidence-based mental healthcare and several reviews have demonstrated small but positive effects on mental health outcomes [24–26]. A recent rapid meta-review of digital mental health interventions delivered to adults during COVID-19 pandemic found evidence to support the usability, safety, acceptability, and effectiveness of these interventions for improving mental health [25]. However, the suitability of digital mental health technologies for addressing student mental health during COVID-19 school closures remains unknown. Based on the Diathesis-stress model, a trial conducted in Australian secondary schools demonstrated that the universal, classroom administration of a web-based cognitive behavioral therapy program during a stressful period can reduce the development and exacerbation of depressive symptoms in students [27]. In New Zealand, secondary school students who received a wellbeing app for six weeks during the COVID-19 pandemic experienced significant improvements in anxiety, depression, stress, and well-being [28]. In the US, school counsellors reported using a range of online tools to address students’ academic, social, and emotional development during school closures [20], but with no evaluation on the effects. As such, it remains unclear what type of psychological approach and digital delivery method is most effective for caring for students’ mental health during a pandemic.

Positive psychology interventions (PPIs) may be ideal for strengthening schools’ response to student mental health during the COVID-19 pandemic as this strengths-based approach targets wellbeing, relationships, and personal growth and is optimized for universal delivery [29–31]. Many of the concepts and principles of positive psychology have been incorporated into disaster response plans, due to the focus on promoting competence, resilience, and self-efficacy [32, 33]. Positive psychology also underpins the positive education approach, a pedagogy familiar to many teachers that emphasizes developing students’ characters and wellbeing skills [34]. As such, PPIs may preserve students’ mental health during school closures and lead to greater resilience and better preparedness for the return to school [35]. Despite the potential suitability, there has been very limited exploration of the utility of a digital PPI for addressing students’ mental health during the COVID-19 pandemic and associated school closures.

### Aims

This paper aimed to explore the short-term mental health impacts of a six-week web-based positive psychology program that was delivered to a sample of secondary school students during the first wave of the COVID-19 pandemic in New South Wales, Australia. Students’ symptoms of anxiety, depression, and help-seeking intentions for mental health were assessed using self-report scales, alongside their perceived changes in mental health status and help-seeking behavior for mental health. This paper also explored the impacts of gender and history of mental illness on changes in mental health during this time given the emergence of these factors in past research [10–12]. Together, these findings may help to determine the suitability of a digital positive psychology approach for preserving students’ mental health during periods of significant disruption.

## Method

### Design and setting

This paper outlines an analysis of a subset of data initially collected as part of cluster-randomized trial that aimed to evaluate the implementation of a digital mental health service in Australian schools. The full trial was postponed due to COVID-19, thus the study presented here is a quasi-experimental design examining the pre and post-test effects of one intervention that was part of this larger trial [36]. The current paper utilizes only the study data collected before and after the COVID-19 school closures in New South Wales, Australia (February 2020 to August 2020). Baseline data was collected prior to school closures and government restrictions (i.e., NSW school term 1: 11th February to 19th March 2020). Australian Federal and State government-mandated restrictions, which included the shift to home-based learning, were implemented on 23rd March 2020. At this time, domestic and international borders were closed, non-essential services were closed, public gatherings were limited to two people and movement outside the home was restricted to essential shopping, medical care, daily exercise or attending employment if unable to do so from home [37]. Schools re-opened for face-to-face teaching in NSW on the 27th May 2020 and many restrictions had been lifted by the end of June 2020. Post-test data were collected after this period and ranged from 20 to 24 weeks post-baseline (i.e., NSW school term 3: 23rd July to 6th August 2020).

### Recruitment, consent, and procedure

Schools were recruited for the cluster-randomized trial through adverts published in various newsletters (i.e., the Black Dog Institute NSW School Counsellors e-newsletter; the NSW School-Link newsletter); the Black Dog Institute’s website and social media channels; academic conference presentations; and correspondence with the NSW Department of Education. School staff listed in the Black Dog Institute’s national school database were also invited to participate via email from the Research Program Manager. Schools were required to provide a letter of consent from the school principal and school counsellor. All the schools included in this paper provided their consent to participate in the cluster-randomized trial between September 2019 and December 2019, prior to the onset of the COVID-19 pandemic in Australia. Students from consenting schools were recruited using a short information video shown to them in class. Students then received a participant information sheet to review with their parents or guardians, distributed by school staff two weeks prior to the baseline assessment. Schools were also instructed to circulate information about the study to parents via their usual channels of communication. An opt-out consent process was used whereby parents or guardians who did not wish for their child to participate in the study were instructed to notify the school or research team prior to the baseline assessment. These students were then unable to take part. Students whose parents had not opted out provided their own individual consent on the day of the baseline assessment using an online form embedded in the web-based study platform. The online consent procedure required all students to complete a 5-item Gillick Competence measure [38] to ensure they understood the terms and conditions of the research study. After fulfilling the consent requirements, students were invited to register to the web-based study platform using their name, gender, age, email, mobile phone number (optional) and creating a username and password. Upon completion of consent and registration, students were directed to the baseline assessment. The post-test assessment was completed via a unique and secure URL that was emailed to students by the study platform. All consent, registration and assessment procedures were undertaken at school under supervision of a research team member and school representative.

### Participants

A total of 26 schools consented to the trial; however, 2 withdrew due to the pandemic and 20 postponed their involvement. This paper includes the 4 remaining schools, 3 of which were in major cities, and one was inner regional. Three were government-funded co-educational and one was non-government funded single-sex (male). A total of 535 students aged 12 to 15 years from the nominated study grades (Grades 8 or 9) were invited to take part. Of these, 64 did not provide personal consent and an additional 26 had their parents or guardians opt-out. A total of 445 students (*n* = 445/535, 83.2%) consented and began the baseline assessment. Of these, 336 (*n* = 336/445, 75.5%) completed post-test.

### Intervention

After completion of the baseline mental health measures, students in the current paper were automatically redirected by the study platform to complete the Bite Back Mental Fitness Challenge [39]. This structured web-based positive psychology program was developed by clinical psychologists at the Black Dog Institute and is freely available to the public [40, 41]. Targeted at youth aged 13 to 16 years, the Bite Back Mental Fitness Challenge guides students through five domains of positive psychology (gratitude, mindfulness, connections, character strengths, meaning and purpose). The program consists of 7 self-directed modules (including an introductory module) that are designed to be completed sequentially over a six-week period. Each week focuses on a separate domain and contains animations, questionnaires and activities that aim to encourage students’ self-reflection and personal growth. Students who complete the modules are automatically eligible to win prizes (e.g. 50AUD gift vouchers) that are offered as completion incentives by the program (not the research team). Throughout challenge, students are encouraged to engage with the Bite Back website [42], which provides more detailed information on wellbeing and activities targeting four additional positive psychology domains (optimism, flow, healthy lifestyles, positive relationships). The website also provides links to other relevant resources and information on where to seek help when faced with a mental health problem. While the Bite Back Mental Fitness Challenge is included as a learning activity in the NSW secondary school Stage 5 Physical Development and Health Education curriculum [43], it was not delivered in this way in the current study. In the current study, students were simply directed to use the program as they wished during school closures. All students received 3 automated reminders to use the program (via SMS and email), sent by the study platform. Students could also configure additional reminders in the Bite Back Mental Fitness Challenge Program.

### Measures

#### Demographics

At baseline, participants provided their age, year group, gender, whether they identified as lesbian, gay, bisexual, transgender, queer (or questioning), and intersex (LGBTQI) and whether they identified as Aboriginal and Torres Strait Islander (yes, no, I’d rather not say).

#### History of mental illness

At baseline, participants were asked whether they had ever experienced a mental health problem or been diagnosed with a mental health illness like anxiety or depression (yes, no, I’d rather not say).

#### Anxiety symptoms

Anxiety symptoms were measured using the Generalised Anxiety Disorder 2-item questionnaire (GAD-2) [44] and the Generalized Anxiety Disorder 7-item questionnaire [GAD-7; 45]. The GAD-2 uses the first two items of the GAD-7 and similarly assesses the frequency of generalised anxiety symptoms over the past two weeks. Both scales use a 4-point likert scale, ranging from “Not at all (0)” to “Nearly every day (3)”. If a score of three or greater was reached on the GAD-2, participants were provided with the full 7-item scale. This was done to reduce burden on participants and ease of administration for delivery in classrooms. In the current study, the total scores correspond to the following levels of symptom severity: ‘nil to minimal’ (0 to 2 on GAD-2), ‘mild’ (3 to 9 on GAD-7), ‘moderate’ (10 to 14 on GAD-7), ‘severe’ (15 + on GAD-7). In this paper, participants who qualified for completion of the GAD-7 were deemed as ‘symptomatic’. The GAD scales have been used in young adolescents, with strong reliability and validity [46]. The Cronbach’s alpha was 0.85 for the GAD-2 and 0.77 for the GAD-7.

#### Depressive symptoms

Symptoms of depression were measured using the Patient Health Questionnaire 2-item questionnaire (PHQ-2) [47] and the Patient Health Questionnaire 8-item [PHQ-8; 48]. The PHQ-2 includes the first two items of the full PHQ scale and similarly assesses the frequency of depressed mood and anhedonia over the past two weeks. Both scales use a 4-point likert scale ranging from “Not at all (0)” to “Nearly every day (3)”. If a score of three or above was reached on the PHQ-2, the full 8-item PHQ was administered. This was done to reduce burden on participants and ease of administration for delivery in classrooms. In the current study, the total scores corresponded to the following levels of symptom severity: ‘nil to minimal’ (0 to 2 on PHQ-2), ‘mild’ (3 to 9 on PHQ-8), ‘moderate’ (10 to 14 on PHQ-8), ‘moderately severe’ (15–19 on PHQ-8) or ‘severe’ (20 + on PHQ-8). In this paper, participants who qualified for completion of the PHQ-8 were deemed as ‘symptomatic’. The PHQ is a reliable and widely validated measure of depression symptoms [α = 0.81–0.87; 48, 49, 50]. The Cronbach’s alpha was 0.68 for the PHQ-2 and 0.82 for the PHQ-8.

#### Help-seeking intentions for mental health

Help-seeking intentions for mental health were measured by the General Help-Seeking Questionnaire [GHSQ; 51]. Participants were asked to rate how likely they were to seek help from 13 sources when having a tough time with their mental health. Items were rated on a 5-point Likert scale ranging from “extremely unlikely” to “extremely likely”. The GHSQ has been widely used in adolescents aged 11 to 18 years and has demonstrated psychometric properties for measuring help-seeking intentions in this age group [52]. A total score was calculated (range: 13 to 61) with higher scores indicative of greater intentions to seek help and Cronbach’s alpha was 0.83.

#### Perceived changes in mental health due to COVID-19

At post-test only, participants were asked to indicate to what degree their mental health and well-being had been impacted by the COVID-19 pandemic (answered ‘a lot better’, ‘a little better’, ‘stayed the same’, ‘a little worse’, ‘a lot worse’). This item has been used in another large youth survey of COVID-19 impacts on mental health [10].

#### Helping-seeking behaviour and sources of help

At post-test only, participants were asked whether they had looked for more information or support for their mental health and well-being since the pandemic (answered yes or no). Participants who responded ‘yes’ were asked to select where they had sought more information or support for their mental health using a ‘check all that apply’ item from a list of nine options (e.g., Internet) and one free response option. Participants were also asked which option(s) they found most helpful by using a single ‘check all that apply’ item from the same list of nine options.

#### Exposure to COVID-19

Exposure to the COVID-19 virus was assessed with a series of Yes/No items that assessed whether the participant (1) had been tested for COVID-19, (2) was awaiting test results for COVID-19, (3) had a family member or close contact had been diagnosed with COVID-19, and (4) had been required to quarantine for 14 days.

#### Program use

Use of Bite Back was measured by the number modules (total of 7) accessed by students throughout the study period.

### Data collection and statistical analyses

Data were collected and stored securely using the Black Dog Institute Research Engine hosted on the UNSW servers in Australia. Data were extracted from the research engine via Microsoft Excel and imported into SPSS version 26.0 (SPSS Inc, Chicago, Il, USA, 2017) for analysis. Descriptive analyses were conducted to describe the sample. The outcomes of interest were group-level changes in GAD-2, PHQ-2 and GHSQ scores from baseline to post-test. These were determined using a mixed-effect model repeated measures (MMRM) analysis. As literature has indicated that COVID-19 may have a greater impact on females [8] and those with a history of mental illness [10, 11], the main MMRM model was repeated with gender and history of mental illness added as covariates. The interaction between time and history of mental illness and time and gender and help-seeking intentions was also examined across the whole sample.

A separate analysis was conducted to examine the changes in GAD-2, PHQ-2 and GHSQ scores from baseline to post-test in the subset of participants who perceived their mental health to have worsened during the pandemic. For this analysis, participants who perceived their mental health as “a little worse” or “a lot worse” were combined and those who perceived their mental health as “a little better” or “a lot better” were combined. Another separate analysis was conducted in the subset of participants who reported that their mental health had worsened during the pandemic *and* who were symptomatic for anxiety or depression at baseline (i.e., reached the threshold for full completion of the GAD-7 or the PHQ-8 questionnaires). The latter group were also examined separately. Further analyses to explore whether there was a significant interaction between time and history of mental illness, as well as time and gender were also conducted in the symptomatic participants only. Symptomatic participants who chose not to disclose their mental health history or who identified their gender as ‘other’ were not included in these analyses due to low numbers. The interaction between time and history of mental illness and time and gender and help-seeking intentions was also examined across the whole sample. In all models, participants were included as a random effect, time as a fixed effect, and school as a random effect to evaluate and accommodate for potential cluster effects. The false discovery rates for p-values in post-hoc tests were corrected to q-values using the Benjamini and Hochberg [53] procedure.

## Results

### Sample characteristics

As outlined in Table 1, the mean age of students was 13 years (SD: 0.53) and most experienced nil to minimal symptoms of anxiety and depression at baseline. In total, 121 (27.6%) were classified as ‘symptomatic’ for anxiety or depression. Very few students had contracted COVID-19 or had been required to self-isolate. The mean number of program modules accessed by participants was 2.31 (SD: 2.38, range: 0–7); however, 30.4% (*n* = 133) did not complete any. Over a third (*n* = 164/438; 37.4%) accessed three or more modules and 14.4% (*n* = 63/438) completed all.

**Table 1:** Participant characteristics of baseline sample (N = 438)

	Mean	SD (Range)	
Age (years)	13.08	0.53 (12 to 15)	
	**N**	**%**	
Gender			
Female	107	24.4	
Male	322	73.5	
Other	9	2.1	
LGBTQI	11	2.5	
Aboriginal or Torres Strait Islander	27	6.2	
History of mental health problems or mental illness	69	15.7	
COVID exposure^a^			
Contracted COVID-19	3	0.9	
Tested for COVID-19	59	16.8	
Family member or close contract contracted COVID-19	16	4.5	
Required to quarantine or self-isolate for 14 days	5	1.4	
Severity of anxiety symptoms^b^			
Nil-minimal	352	80.4	
Mild	23	5.3	
Moderate	30	6.8	
Severe	33	7.5	
Severity of depressive symptoms^c^			
Nil-minimal	357	81.5	
Mild	19	4.3	
Moderate	25	5.7	
Moderately-severe	21	4.8	
Severe	16	3.7	
Symptomatic at baseline	121	27.6	
Reached threshold for both scales	46	10.5	
Reached threshold for GAD-2 only	40	9.1	
Reached threshold for PHQ-8 only	35	7.9	

^a^ COVID exposure variables *n* = 352 due to survey administration at post assessment time point

^b^Symptom severity for anxiety based on total scores: ‘nil to minimal’ (< 3 on GAD-2), ‘mild’ (3 to 9 on GAD-7), ‘moderate’ (10 to 14 on GAD-7), ‘severe’ (15 + on GAD-7)

^c^Symptom severity for depression based on total scores: ‘nil to minimal’ (< 3 on PHQ-2), ‘mild’ (3 to 9 on PHQ-8), ‘moderate’ (10 to 14 on PHQ-8), ‘moderately severe’ (15 to 19 on PHQ-8), ‘severe’ (20+)

### Changes in symptoms of anxiety, depression, and helping-seeking intentions

Table 2 outlines the mean symptom scores for anxiety, depression and help-seeking intentions at baseline and post-test. When examining the total sample, there were no significant changes in anxiety (GAD-2) [*F*(1, 76.01) = 0.01, *p* = .921], depressive (PHQ-2) symptoms [*F*(1, 52.94) = 0.021, *p* = .884] or help-seeking intentions [*F*(1, 378.35) = 0.348, *p* = .556] between baseline and post-test. Similarly, there were no significant changes in anxiety (GAD-2, *p* = .856), depressive symptoms (PHQ-2, *p* = .489) or help-seeking intentions (*p* = .547) between baseline and post-test when gender and history of mental illness were controlled for.

**Table 2:** Mean and standard deviations for continuous outcome measures at baseline and post-test in whole sample and selected subsets

Measure	Sample subset	Baseline			Post-test			
		*n*	Mean	SD	*n*	Mean	SD	MD
GAD-2	Total sample	438	1.23	1.73	336	1.21	1.68	− 0.02
	Perceived worsened mental health^a^	97	2.02	1.96	95	2.35	1.95	+ 0.33*
	Perceived better mental health^b^	52	0.96	1.58	51	0.86	1.33	− 0.10
	Perceived no change in mental health	203	0.84	1.40	190	0.74	1.32	− 0.10
PHQ-2	Total sample	438	1.21	1.53	340	1.25	1.44	+ 0.04
	Perceived worsened mental health^a^	97	1.93	1.70	95	2.35	1.63	+ 0.42**
	Perceived better mental health^b^	52	0.87	1.16	51	0.67	0.86	− 0.20
	Perceived no change in mental health	203	0.91	1.33	194	0.86	1.15	− 0.05
GHSQ	Total sample	438	34.53	8.60	341	34.37	9.53	− 0.16
	Perceived worsened mental health^a^	97	33.76	8.60	95	31.82	9.32	-1.94
	Perceived better mental health^b^	52	35.12	9.13	51	36.27	10.58	+ 1.15
	Perceived no change in mental health	203	35.16	8.34	195	35.11	9.14	− 0.05
	Symptomatic at baseline	121	32.81	8.45	90	31.42	9.55	-1.39
	No history of mental illness	338	35.14	8.47	269	34.82	9.67	− 0.32
	History of mental illness	69	33.22	8.86	50	32.54	8.49	− 0.68
	Male	322	34.58	8.71	249	34.72	9.97	+ 0.14
	Female	107	35.24	7.76	84	33.81	8.36	-1.43
GAD-7	Symptomatic at baseline^c^	121	9.52	6.65	89	7.45	7.03	-2.07
	No history of mental illness	64	7.61	6.26	50	5.90	6.66	-1.71
	History of mental illness		12.21	6.52	28	9.43	7.32	-2.78
	Male	64	7.89	6.63	47	5.64	6.71	-2.25
	Female	56	11.25	6.25	41	9.24	6.83	-2.01
PHQ-8	Symptomatic at baseline^c^	121	9.41	7.38	90	7.53	7.87	-1.88
	No history of mental illness	64	6.53	5.99	50	6.22	7.33	− 0.31
	History of mental illness	39	13.13	7.85	29	10.00	8.50	-3.13
	Male	64	8.78	6.60	48	6.58	7.62	-2.2
	Female	56	15.38	5.35	41	8.24	7.82	-1.65

*Note.* GAD-7 and PHQ-8 were only administered to the subset of participants who exceeded the threshold of 3 on the GAD-2 and PHQ-2 at baseline (i.e., were symptomatic). Significant Mean Differences (MD) are denoted by *p < .05, ** *p* < .01

^a^ Participants who reported that their mental health was ‘a little’ or ‘a lot’ worse since the pandemic

^b^ Participants who reported that their mental health was ‘a little’ or ‘a lot’ better since the pandemic

^c^ Participants who qualified for the full GAD-7 or PHQ-8 scales at baseline

### Changes in mental health and help-seeking outcomes in participants who were symptomatic at baseline stratified by history of mental illness and gender

In the participants who were symptomatic at baseline (*n* = 121), no significant changes in, or interaction between, time and history of mental illness for anxiety [*F*(1, 55.8) = 0.47, *p* = .496] or depressive symptoms [*F*(1, 43.3) = 2.04, *p* = .160] were found. There was also no significant interaction between, time and history of mental illness for help-seeking intentions [*F*(1, 88.1) = 0.95, *p* = .331]. Similarly, there were no significant interaction between time and gender for anxiety [*F*(1, 53.0) = 0.38, *p* = .543], or depressive symptoms [*F*(1, 41.8) = 2.24, *p* = .142]. Lastly, there were no significant changes in, or interaction between, time and gender for help-seeking intentions in this subset [*F*(1, 87.7) = 2.11, *p* = .150].

### Perceived changes in mental health and help-seeking behavior for mental health

As outlined in Table 2, 27.5.% (*n* = 97/352) of the sample perceived that their mental health had worsened during this period of the pandemic, 14.8% (*n* = 52/352) perceived that their mental health had improved, and 57.7% (*n* = 203/352) perceived that their mental health had stayed the same. At post-test, 7.7% (*n* = 27/352) reported that they had looked for more mental health information and support during this period. In these participants, the most common sources of mental health support were the Internet (77.8%, *n* = 21/27), parents (55.6%, *n* = 15/27), and friends (37.0%, *n* = 10/27). The most helpful sources of support were the Internet (63.0%, *n* = 17/27), parents (48.1%, *n* = 13/27) and friends (18.5%, *n* = 5/27).

A significant interaction was found between perceived mental health and time for anxiety symptoms [*F*(2, 337.7) = 3.68, *p* = .002]. Post-hoc tests showed that GAD-2 scores significantly increased from baseline to post-test among participants who perceived their mental health to have worsened (*t*(336) = 2.49, *p* = .022, *q* = 0.022, *d*=-0.36, 95% CI=[-0.65, -0.08]). There was no change in GAD-2 scores among participants who perceived their mental health to have improved (*t*(335) = 0.60, *p* = .549) or had stayed the same (*t*(340) = 0.97, *p* = .334). Similarly, a significant interaction was found between change in perceived mental health and time for depressive symptoms [(*F*(1, 339.5) = 4.66, *p* = .010]. Post-hoc tests showed that PHQ-2 scores significantly increased among participants who perceived their mental health to have worsened (*t*(341)=-3.02, *p* = .003, *q* = 0.005, *d*=-0.44, 95% CI=[-0.72, -0.15]). There was no change in PHQ-2 scores among participants who perceived their mental health to have improved (*t*(340) = 0.92, *p* = .357) or had stayed the same (*t*(344) = 0.49, *p* = .661). No significant interaction effect was found for time and perceived level of change on mental health on help-seeking intentions [*F*(2, 343.0 = 2.48, *p* = .084)]. Among the subset of participants who perceived their mental health to have worsened and who were symptomatic at baseline (*n* = 45), there were no significant changes in or interaction effects for time and perceived level of change on anxiety [*F*(1, 32.0) = 1.35, *p* = .253] or depressive symptoms [*F*(1, 26.6) = 0.86, *p* = .361].

## Discussion

The current paper explored the potential benefits of a web-based positive psychology program that was delivered universally to secondary school students during school closures in the first wave of the COVID-19 pandemic in Australia. This paper utilized data that was initially collected as part of another cluster randomized trial and was instead used to examine whether a structured web-based positive psychology program could support students’ mental health during this disruptive time. This investigation was of timely importance, as many schools struggled to address student mental health remotely. The findings indicated that at the group-level, anxiety and depressive symptoms among students who received the program did not significantly change over the study period. There was also no significant change in students’ intentions to seek help for their mental health. Although there was a mean reduction in anxiety and depressive symptoms among the subset of students who were symptomatic at baseline, this change was not significant. In contrast to past studies [10, 11], no significant interaction effects were found for gender or history of mental illness. While the findings do not show support for the program’s effectiveness at lowering symptoms in the short-term when delivered universally to students, the program may have been beneficial for preventing deterioration in these measures. Given the negative mental health impacts of the COVID-19 pandemic and associated school closures, the universal delivery of a web-based positive psychology program may have helped to slow the onset of anxiety and depressive symptoms in students. The program may also show greater promise for selected delivery to students with symptoms. Longer follow-up of these individuals, with a control comparator, is required to determine the suitability of this approach for schools wishing to address student mental health in times of national and international crisis.

While there was no significant change in symptom scores for anxiety and depression across the whole sample, one in four students perceived that their mental health had worsened during this initial period of the pandemic. Notably, these students were found to experience a significant increase in their symptoms of anxiety and depression at post-test. These rates are significantly lower than those found in Li et al’s study [10], where 75% of Australian teens reported that their mental health had worsened and almost half had levels of distress that corresponded to clinical illness. However, these latter results were collected between June to August in 2020, when young people had been exposed to the pandemic for longer. Together, these findings indicate that students who perceived their mental health to have worsened are an ‘at risk’ group warranting special attention. The delivery of a positive psychology program alone to these students may have been inadequate and more intensive psychological intervention was required. A simple 1-item measure, like perceptions of mental health change, may therefore be an important indicator of mental health status and need rather than symptom measures alone. This notion has been reflected in other large population studies of mental health. For example, Chiu and colleagues [54] found the prevalence of depression and psychological distress as determined by validated symptom scales, remained unaltered over time, but the number of individuals who perceived ‘fair’ or ‘poor’ mental health status increased. Similarly, Simpson and colleagues [55] also found that while the prevalence of depression remained stable, self-reported diagnoses of mood disorders increased. While the accuracy of perceived mental health status for predicting need for care in adolescents requires further validation, the simplicity of such an item may represent significant advantages for schools wishing to easily identify the students who require additional support. Furthermore, the perceived mental health change experienced by this subset of participants may have been linked to broader effects of the pandemic, which may have been better captured in additional measures of common and transitory responses to crisis (e.g. well-being, social support, life satisfaction, health anxiety, fear, distress, rage, emotional and behavioural dysregulation, functioning), rather than symptoms alone [12, 56]. Results from this study indicate that effective mental health surveillance in schools, particularly in times of crisis, requires the use of several measures. Future studies should examine a broader range of mental health outcomes, especially when these outcomes are being used to determine the need for, or effects of, intervention.

### Limitations

Given the secondary nature of this study, there are several limitations that impacted the findings. Firstly, the lack of significant changes in symptoms of anxiety and depression in the total sample is likely to have been influenced by floor effects, given most students reported nil to minimal symptoms at baseline. Low levels of symptoms may have also limited help-seeking behaviour and intentions, as many students did not have a need for mental health support. This is a common challenge with school-based mental health trials [57], particularly when the psychological interventions being examined have only small effects when delivered universally. Larger sample sizes are therefore needed to account for the absence of symptoms in students [58]. Further, as the current study did not include a control or comparison group, we cannot definitively attribute any stabilisation or improvement of symptoms to the universal positive psychology program.

While anxiety and depression are common illnesses among adolescents, and associated with significant school disruption, the findings of this study may have been limited by the focus on these outcomes. Given the holistic nature of the positive psychology program, broader measures of wellbeing may have been more suitable for determining the impacts of the intervention on students. These may include, but are not limited to, absenteeism, motivation, engagement in learning, and school connectedness. While a strength of the current study is the number of adolescent male participants, biased samples have been documented as a key concern in COVID-19 research [59]. Different results may be found in samples including more female students, as adolescent females often report higher rates of anxiety and depression than males [60]. Different results may also be found in students from schools located in different areas of Australia or schools that experienced different levels of disease exposure or restrictions. Finally, the current study only captured the initial acute phase of the pandemic crisis in Australia, and it remains unclear what the longer-term impact on mental health and help-seeking will be. Given the rapidly evolving situation with COVID-19 globally, ongoing studies will help to better understand the short- and longer-term impacts of this global issue.

### Implications for design and development of school-based digital mental health programs

This study presents several key considerations for researchers wishing to Capitalize on digital mental health programs for remote delivery in schools. While a publicly accessible, evidence-based program like the Bite Back Mental Fitness Challenge appeared to be ideal for this purpose, this study indicated that achieving high rates of module completion remained a challenge. Given the variation in module completion, the results suggest that the self-directed content may not be appropriate for all students, particularly when school is operating remotely. Future interventions could overcome this by embedding the intervention content into class activities or homework and sending reminders with other school-related content or from the school itself. However, as school closures introduce more stressors to the already over-burdened role of teachers, owners of these mental health programs may need to supplement their delivery with externally provided support. The increased availability of video-conferencing due to COVID-19 could be capitalized on to provide this. Parents could also be engaged to encourage program completion at home; however, additional information and strategies would be needed to support this. It is often challenging to interpret non-completion rates of universal digital mental health programs, as many students may disengage due to feeling better, or because the program failed to target their needs. It is also unclear whether the delivery of such programs creates a ‘safety net’ for students, whereby they may not directly engage with the content but feel more secure knowing it is available when in need. Alternatively, students’ low completion rates may be related to concerns about their own physical health or the health of family members due to COVID-19 [61], or to changes in school workloads due to the shift to remote learning from home, leaving less time to engage in a digital program. Future work should aim to examine these effects of remotely delivered digital programs while also assessing any indirect changes in the provision of school-based supports that may influence student outcomes. Lastly, researchers and program developers would benefit from greater consideration of the varied contexts and circumstances in which their programs can be delivered. As school closures were abrupt, programs that can be quickly and easily delivered, as well as segmented or adapted to include external support are likely to have greater reach and benefits on student mental health.

## Conclusions

The COVID-19 pandemic has had significant impacts on the mental health of students worldwide. When delivered universally, the web-based positive psychology program examined in our study did not appear to be associated with group-level reductions in symptoms of anxiety or depression. The program may have potential for preventing the deterioration of mental health measures in students during times of crisis or when selectively delivered to students with symptoms. Larger studies with more complex designs are needed to confirm this. Our findings strongly suggest that mental health surveillance in schools, particularly during periods of remote learning and heightened stress, should utilize multiple measures of mental health outcomes, including perceived mental health change, to identify students who require additional support. These findings confirm the importance of evaluating psychological interventions delivered to students during the pandemic and may help inform future interventions aimed at supporting the mental health of secondary school students, especially during times of crisis.

## Data Availability

Not publicly available due to the sensitive nature of the data and ethical guidelines.
